# Altered representation of facial expressions after early visual deprivation

**DOI:** 10.3389/fpsyg.2013.00878

**Published:** 2013-11-21

**Authors:** Xiaoqing Gao, Daphne Maurer, Mayu Nishimura

**Affiliations:** ^1^Centre for Vision Research, York UniversityToronto, ON, Canada; ^2^Department of Psychology, Neuroscience & Behaviour, McMaster UniversityHamilton, ON, Canada

**Keywords:** facial expressions, emotion, experience, visual deprivation, multi-dimensional scaling, sleeper effect

## Abstract

We investigated the effects of early visual deprivation on the underlying representation of the six basic emotions. Using multi-dimensional scaling (MDS), we compared the similarity judgments of adults who had missed early visual input because of bilateral congenital cataracts to control adults with normal vision. Participants made similarity judgments of the six basic emotional expressions, plus neutral, at three different intensities. Consistent with previous studies, the similarity judgments of typical adults could be modeled with four underlying dimensions, which can be interpreted as representing pleasure, arousal, potency and intensity of expressions. As a group, cataract-reversal patients showed a systematic structure with dimensions representing pleasure, potency, and intensity. However, an arousal dimension was not obvious in the patient group's judgments. Hierarchical clustering analysis revealed a pattern in patients seen in typical 7-year-olds but not typical 14-year-olds or adults. There was also more variability among the patients than among the controls, as evidenced by higher stress values for the MDS fit to the patients' data and more dispersed weightings on the four dimensions. The findings suggest an important role for early visual experience in shaping the later development of the representations of emotions. Since the normal underlying structure for emotion emerges postnatally and continues to be refined until late childhood, the altered representation of emotion in adult patients suggests a sleeper effect.

## Introduction

Emotional facial expressions are an important channel of nonverbal communication. Both the ability to express one's feelings through facial expressions and the ability to perceive other people's emotional states through decoding of their facial expressions are crucial for smooth social interactions. Previous studies have suggested a biologically prepared system that can generate and decode different types of facial expressions (e.g., Ekman and Friesen, 1971; Izard et al., 1980). Congenitally blind adults are able to voluntarily generate the same types of facial expressions as typical sighted adults, despite never having had any visual experience (Galati et al., 1997; Matsumoto and Willingham, 2009; see Tröster and Brambring, 1993 for similar data from infants). Adults blind from an early age, who had minimal experience with facial expressions early in life, have brain responses to haptic input of facial expressions that are similar to those of typical sighted adults (Kitada et al., 2013). However, visual experience seems to be necessary to fine-tune the mental representation of facial expressions, as the facial expressions posed by the congenitally blind adults are poorly recognized by typical sighted adults (Galati et al., 1997).

Cross-cultural similarity in the production and recognition of some facial expressions, which have come to be known as the six basic expressions (Ekman et al., 1969; but see Biehl et al., 1997; Elfenbein et al., 2007; Jack et al., 2009, 2012, for evidence on cultural differences) suggests that the postnatal refinement is likely to operate in an experience-expectant manner (Leppänen and Nelson, 2008). However, there can be experience-dependent alterations if the individual receives atypical exposure to certain facial expressions in the rearing environment. Physically abused children, who are likely to see angry faces more often than typical children, have a lower threshold than age-matched typical children to detect anger (Pollak et al., 2000; Pollak and Kistler, 2002; Pollak and Sinha, 2002; Pollak and Tolley-Schell, 2003; Shackman et al., 2007). Neglected children, who are likely to have less experience with faces overall, are less accurate than age mates in discriminating among expressions (Pollak et al., 2000). Similar to neglected children, children who spent their early life in Romanian orphanages, where they had relatively little social interaction with adults, are less accurate in identifying facial expressions or matching facial expressions to verbal scenarios (Wismer Fries and Pollak, 2004). However, the timing of susceptibility of the facial expression system to atypical experience is unclear.

Previous studies suggest visual experience early in life is especially important for the later development of face processing skills. With a rare sample of patients treated for congenital cataracts, our group has found that despite treatment during infancy, even as adults such patients show deficits in recognizing faces with a changed viewpoint, in discriminating two faces differing only in the location of the internal features, and in recognizing famous or recently learned faces (Le Grand et al., 2001; Geldart et al., 2002; Robbins et al., 2010; de Heering and Maurer, 2012). Their patterns of aftereffects suggest that their underlying representations of faces are also not normal (Robbins et al., 2012). Many of these face processing skills emerge postnatally and continue to develop throughout childhood. Therefore, the later deficits in patients suggest that patterned visual input early in life plays a role in allocating neural resources for the processing of facial identity that are necessary not for perception during early infancy, but rather for later refinement.

The development of sensitivity to facial expressions shares many common features with the development of sensitivity to facial identity. Both have an early onset of sensitivity (e.g., facial expression: Field et al., 1982; Farroni et al., 2007; facial identity: Field et al., 1984; Bushnell et al., 1989; Pascalis et al., 1995) and a long developmental course (Camras and Allison, 1985; Kolb et al., 1992; Markham and Adams, 1992; Gosselin and Larocque, 2000; De Sonneville et al., 2002; Mondloch et al., 2003a; Durand et al., 2007; Thomas et al., 2007; Herba et al., 2008; Gao and Maurer, 2009, 2010; Montirosso et al., 2010). It is possible that early visual input may also play an important role in allocating neural resources for the later development of normal sensitivity to facial expression. On the other hand, by adulthood, the neural systems for facial identity and facial expression are largely separate (Haxby et al., 2000) and unlike identity, the representation of facial expressions is multimodal. Therefore, it may be less susceptible to early visual deprivation than is the case for facial identity, since input from other sensory modalities such as hearing and touch, may drive normal development despite the lack of visual input before treatment.

In the only study to date, the processing of facial expressions appeared to be spared after early binocular visual deprivation: patients (tested when they were at least 10.7 years old, at least 10.5 years after treatment) were as accurate as controls in matching facial expressions across changes in identity (Geldart et al., 2002). However, this study investigated only the recognition of intense facial expressions of happiness, surprise, disgust, plus neutral. Typical adults cannot only divide facial expressions into discrete categories, but they also perceive the interrelationship among facial expressions (Russell and Bullock, 1985). A previous study found abnormalities in judging those interrelationships in congenitally blind adults (Galati et al., 1997), who represented disgust and happiness in close proximity, a pattern that is not seen in typical adults, whether tested haptically or visually. In addition, studying the interrelationship among facial expressions can at the same time provide information about categorization of facial expressions (Goldstone, 1994).

The interrelationships among facial expressions have been identified in typical adults by asking them to rate the similarity of emotions and then using the statistical technique of multi-dimensional scaling (MDS) to find the most parsimonious underlying structure that fits the data. From such MDS, it has been inferred that typical adults represent the perceived similarity of emotions in a structure with at least four dimensions. Inspection of the expressions located at the opposite ends of each dimension allowed researchers to infer that they represent pleasure (differentiating happy from the negative emotions), arousal (differentiating high arousal emotions like fear or anger from low arousal emotions like sadness), intensity (representing lower and higher intensities of the same expression), and potency (differentiating emotions that make one feel empowered—like anger—from emotions that make one feel weak—like fear) (Plutchik, 1980; Russell, 1980; Russell and Bullock, 1985, 1986; Fontaine et al., 2007; Gao et al., 2010). Complementary hierarchical clustering analyses revealed that the lowest level of clustering grouped the different intensities of the same expression (except for some low intensity ones that are grouped with neutral), and that higher levels grouped expressions into (happiness, neutral, fear, and surprise), (sadness, disgust, and anger). This structure begins to emerge very early, with pleasure and arousal dimensions evident as early as age 2 (Russell and Bullock, 1986), but even at age 14, the structure is not fully adult-like (Gao et al., 2010). In the current study we used these techniques to measure the multidimensional structure underlying the perceived similarity among emotions in cataract-reversal patients. Specifically, we asked whether early visual experience is necessary for an adult-like structure to be built up subsequently during development, or whether early visual deprivation instead leads to the representation of emotions in an altered structure, as it does for facial identity. This approach allowed us to deduce the inherent structuring of emotion relative to each other (e.g., is neutral grouped with an emotion other than happy? is anger rated as highly similar to fear or sadness?). In addition, it allowed us to assess whether, as has been known for visually normal adults since the time of Fechner (Fechner, 1860; von Helmholtz, 1867), the similarity ratings followed Weber's law such that two highly intense expressions (e.g., 70 and 90% intensity) are perceived as more similar than two less intense expressions with the same physical discrepancy (e.g., 50 and 70%). Such relationships among different intensity levels within the same expression wouldn't be revealed by a categorization task.

Adults treated for bilateral congenital cataracts during infancy were compared to an adult control group on the perception of the six basic emotions, each at three intensities, plus neutral. They were shown two faces at a time, and asked to rate the similarity of the two emotions on a 7-point scale. From these similarity ratings, we used MDS to derive the underlying structure for each group.

## Methods

### Participants

We tested 10 adult patients (mean age = 22.0 ± 5.7 years, range = 17–32 years, 4 females) who had dense central bilateral congenital cataracts that prevented patterned visual input until the cataracts were removed surgically during infancy. The patients were then given a compensatory optical correction, usually contact lenses. Details about the patients and their basic vision are summarized in Table 1. For comparison, we also tested 10 young adults (mean age = 18.6 ± 1.6 years, range = 17–22 years, 5 females) with normal vision (Snellen acuity of 20/20 or better in each eye). Because all the patients and the controls were White Caucasian and grew up in Canada, their ratings of the Caucasian faces would not have been affected by any other race effect (Feingold, 1914). The study was approved by the research ethics boards at McMaster University and The Hospital for Sick Children, Toronto. All participants provided informed consent.

**Table 1:** **Details of the 10 patients treated for bilateral congenital cataract**.

**Patient sex**	**Age at test (years)**	**Secondary visual problems^a^**	**Linear letter acuity^b^**		**Duration of deprivation (days)^c^**	
				
			**Right**	**Left**	**Right**	**Left**
F1	15.2	Glaucoma, nystagmus, strabismus surgery	20/63	20/25	92	92
M1	16.9	Glaucoma, nystagmus	20/40	CF3′	106	106
M2	18.0	Glaucoma, nystagmus	20/32	20/32	48	48
M3	18.2	Nystagmus, strabismus surgery	20/40	20/70	100	100
M4	18.3	Glaucoma	CF10′	20/40	65	65
F2	21.3	Glaucoma, nystagmus, strabismus surgery	20/200	20/125	134	134
M5	23.3	Glaucoma, nystagmus	20/80	20/125	97	97
F3	24.2	Nystagmus, strabismus surgery	20/25	20/64	91	91
F4	32.0		20/50	20/63	129	129
M6	32.1	Nystagmus, strabismus surgery	20/125	20/25	181	294

a Secondary visual problems are ones that arose as a result of the initial visual deprivation. In every case, glaucoma was controlled by drugs so that there was no damage to the retina.

b Best corrected linear letter acuity on the day of testing. CF3(10)′ stands for counting fingers at 3(10) feet.

c From birth until the fitting of a compensatory contract lens after surgical removal of the cataract.

### Stimuli

The stimuli were the same as those used in our previous study comparing the underlying structure of children's and adult's perception of similarity among facial expressions (Gao et al., 2010). Specifically, we selected seven photographs of one female model showing intense facial expressions of the six basic emotions (happiness, sadness, fear, anger, disgust, and surprise) and neutral from the NimStim Face Stimulus set (Model 03, Tottenham et al., 2009). The chosen photographs received high agreement on the posed expressions and high ratings of intensity from adults in a separate study (Palermo and Coltheart, 2004). We created three levels of intensity (50, 70, and 90%) for each expression by morphing the original emotional face with the neutral face (for details, see Gao and Maurer, 2009). The intensity was measured as a proportion of the physical difference between neutral and the endpoint of each expression. Note, however, that despite the similar ratings of the endpoints for the six expressions, as in all other studies of facial expressions, we cannot be sure that the endpoints of each expression were equally intense, relative to the strongest possible expression, and hence we cannot be sure that our intermediate steps are equal across expressions.

There were 19 images (6 expressions × 3 intensity + 1 neutral). Stimuli were presented on a 15-inch Macbook pro using custom software. Each face was 6.1 cm wide by 11.0 cm high, or 5.8 by 10.5° of visual angle when viewed from a distance of 60 cm.

### Procedure

**Figure 1:**
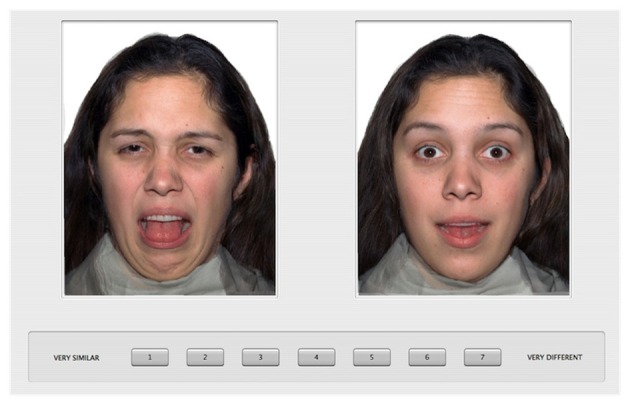
**An example trial of the similarity rating paradigm**.

The procedure was the same as that used previously (Gao et al., 2010). Specifically, the participants were introduced to the task with 20 practice trials during which they rated the similarity of pairs of emotional faces from a male model. A 7-point scale was displayed under the two face images with “1” labeled as “very similar” and “7” labeled as “very different.” To specify the basis for the similarity judgments (Medin et al., 1993), we instructed the participants to give similarity ratings based on the “feeling” that was being portrayed by the individual in the photographs and encouraged them to use the full range of the rating scale. In the testing session, each participant rated the similarity of all possible pairs of the face images from the female model. On each trial, a pair of face images was displayed side by side on the computer screen (see Figure 1 for an example trial). There were a total of 171 pairs, which were displayed in a random order for each participant, and left/right position was also randomized within each pair. The task took about 20 min to complete, and was self-paced.

### Analysis

#### Similarity ratings

**Figure 2:**
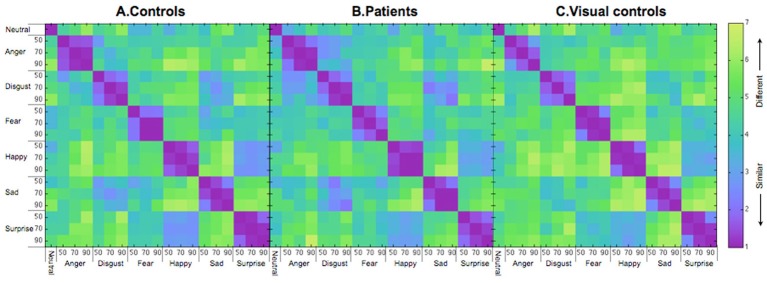
**Similarity matrices for (A) controls, (B) patients treated for congenital bilateral cataracts, and (C) visual controls.** The numbers on the axes represent intensity levels (%). The similarity scores go from 1 (most similar) to 7 (most different).

Figure 2 shows the similarity ratings of the 171 pairs of facial expressions for each group (A. controls and B. patients), averaged across participants. Before using MDS analysis on the group data, we assessed the similarity ratings within each group (patients and controls, separately) and between the two groups. To assess within group similarity, we first randomly selected 10 data sets (1 × 171 similarity vectors) with replacement before splitting the sample into two equal halves. We then computed an average similarity vector for each half of the data before computing a Pearson correlation (r) between them. We conducted 1000 bootstrap samples and estimated the mean within-group correlation and 95% confidence intervals for each group (see Efron, 1979 for more details). We followed the same procedure for the between group analysis except that each correlation was based on half of the patient data and half of the control data.

The mean correlation for similarity judgments by the control group was 0.90 (95% *CI* = 0.79–0.97), which is significantly higher than the mean for the between-group correlation (mean = 0.77, 95% *CI* = 0.62–0.86, *p* < 0.05), but not different from the within-group patient correlation (mean = 0.80, 95% *CI* = 0.55–0.95, *p* = N.S.). The lower between-group correlation than the within (control) group correlation suggests that at a group level the patients' pattern differed from that of the controls. However, the fact that the within (patient) correlation is not different from the between-group correlation (possibly as a result of higher variability within the patients as suggested by the wider confidence interval of the patient group than the control group) weakens the evidence from the correlations that the control group is different from the patient group.

To explore the nature of any differences between the patients and the controls at a group level, we used MDS and hierarchical cluster analysis.

#### Multi-dimensional scaling

**Figure 3:**
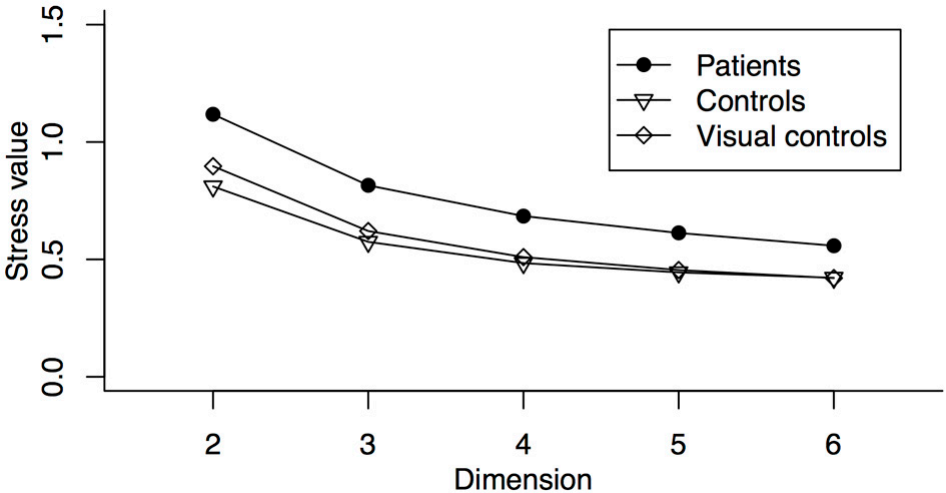
**Stress values for 2- to 6-dimensional solutions for the patients and the controls**.

In order to compare the patients and the controls at a group level without disregarding the individual differences within each group, we used a metric INDSCAL procedure to calculate the MDS solutions. INDSCAL calculates a group solution while modeling individual differences as weightings on different dimensions. To determine the optimal number of dimensions, we calculated the stress values (Kruskal and Wish, 1978) for 2- to 6-dimensional solutions for the patient and the control groups separately (Figure 3). Lower stress values indicate a better fit to the data. As shown in Figure 3, patients had higher stress values than the controls, a pattern suggesting more variability among the patients. For both groups, the stress values reached asymptote around 4 dimensions and at that level the fits for both groups were within the range considered good (Kruskal and Wish, 1978). Since the reduction of stress value was small beyond 4 dimensions while more than 4 dimensions would make the solutions difficult to interpret, we decided to use 4-dimensional solutions for both groups. To provide a complementary view of the similarity judgments to the MDS solutions, we also ran hierarchical clustering on similarity ratings.

## Results

### Dimensionality

**Figure 4:**
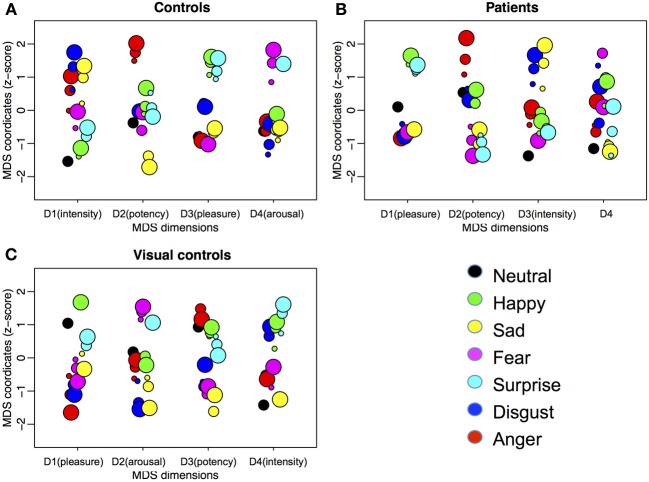
**Four-dimensional MDS solutions for (A) controls, (B) patients treated for congenital bilateral cataracts, and (C) visual controls.** We ordered the dimensions by the amount of variance explained by them from high (D1) to low (D4). Each dimension is plotted in isolation with the horizontal locations of the dots jittered to reduce overlapping. Emotion categories are represented by different colors. Intensities of the emotions are represented by different sizes (small, medium, and big for 50, 70, and 90%, respectively). The interpretations of the meaning of the dimensions are in parentheses.

Figure 4 shows the MDS solutions for the control group (Figure 4A) and the patient group (Figure 4B). We ordered the dimensions by the amount of variance explained by them from high (D1) to low (D4). Because of the intrinsic difficulty in presenting a four-dimensional structure, here we plotted each dimension in isolation. The MDS solution determines the placement of each expression on each of the dimensions. However, the MDS solution does not define the meaning of the dimensions. Previous studies suggest four underlying dimensions of the multidimensional structure of emotions, namely, pleasure, arousal, potency, and intensity (e.g., Russell, 1980; Russell and Bullock, 1985, 1986; Fontaine et al., 2007; Gao et al., 2010). To understand how the dimensions of the current MDS solutions relate to the four dimensions identified in the previous studies, we asked a new group of ten Caucasian adults (mean age = 21.5 ± 4.4 years, range = 17 to 32 years, 6 females) to rate each of the 19 faces on the four dimensions: (1) pleasure, introduced to the raters as a feeling of pleasant or unpleasant; (2) arousal, introduced to the raters as a feeling of aroused/excited/activation or passive/sleep/deactivation; (3) potency, introduced to the raters as a feeling of high control/power or low control/power; and (4) intensity, introduced to the raters as the intensity of feelings expressed on the faces. The participants gave ratings on the 19 faces in a blocked manner, so that within each block, they only gave ratings on one of the four dimensions. The order of the dimensions was randomized. The order of the faces within each block was also randomized. We calculated the correlations between the mean ratings for the faces across participants on the four dimensions and the coordinates of the faces in the MDS solutions (Table 2). We identified the MDS dimension that had the highest and statistically significant loading on each of the rated dimension as representing the rated dimension (Fontaine et al., 2007). We identified dimensions of pleasure (dimension 3), arousal (dimension 4), potency (dimension 2), and intensity (dimension 1) in the controls. However, only the dimensions of pleasure (dimension 1) and potency (dimension 2) were identified in the patients. Although the correlations with the independent ratings did not reveal an intensity dimension in the patients, it is possible the ratings captured the perceived intensity within the set of all 19 faces, but the MDS solution may only capture the relative intensity within each emotion category. The third dimension of the patients' MDS solution may capture the within emotion category intensity, as the intensities increase within most of the emotion categories as a face moves away from neutral. In fact, even for the controls, we can see the within emotion category intensity mapped on the first dimension.

**Table 2:** **Correlations between independent ratings on pleasure, arousal, potency, and intensity and MDS solutions**.

**Rating**	**MDS solutions**											
	**Patients**				**Controls**				**Visual controls**			
	**D1**	**D2**	**D3**	**D4**	**D1**	**D2**	**D3**	**D4**	**D1**	**D2**	**D3**	**D4**
Pleasure	**0.96**	−0.18	−0.54	−0.08	−0.82	0.13	**0.84**	0.37	**0.84**	0.48	0.47	0.61
Arousal	0.44	−0.05	−0.56	−0.32	−0.33	0.55	0.39	**0.72**	−0.08	**0.67**	0.47	0.58
Potency	0.45	**0.64**	−0.38	0.01	−0.38	**0.81**	0.38	−0.09	0.24	0.10	**0.92**	0.31
Intensity	−0.26	−0.06	0.42	−0.05	**0.64**	0.18	−0.09	0.21	−0.56	−0.12	−0.17	0.16

For each rated dimension, the highest and statistically significant (p < 0.05, corrected for family-wise type I error) loading is in boldface.

### Weighting

For controls, as in our previous study (Gao et al., 2010), two dimensions are weighted similarly by the 10 participants, with a bit more scatter in the weighting of the other two dimensions. In contrast, the weightings on all four dimensions vary among the 10 patients. This variability is consistent with their higher stress values for the multidimensional scaling solution than controls and with their wider confidence intervals for the within group correlations. All three measures suggest more variability among the patients than the controls.

### Clustering

**Figure 5:**
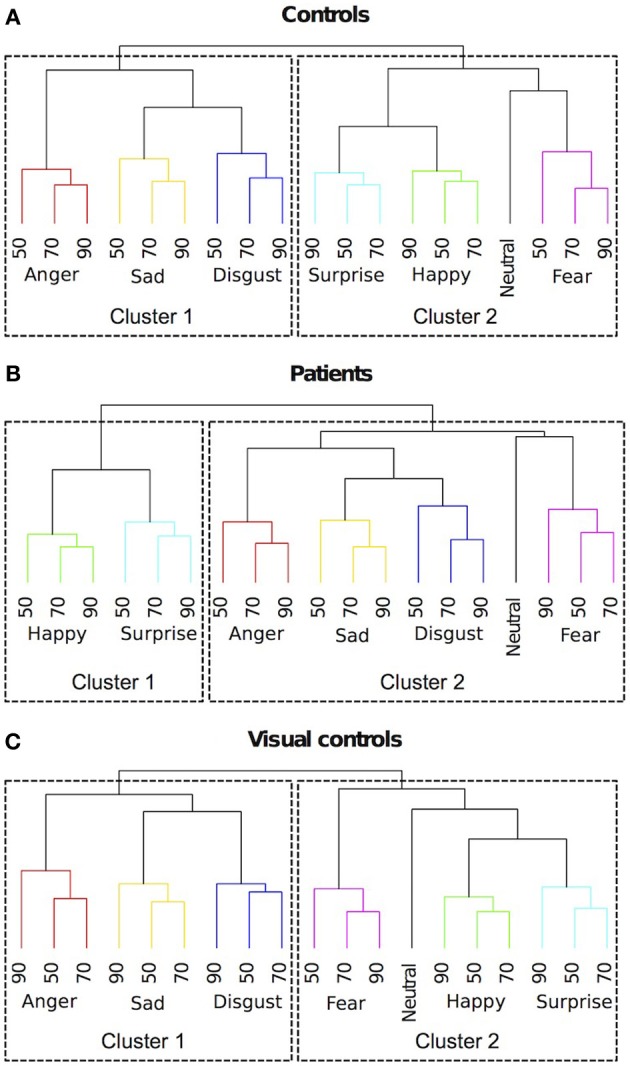
**Dendrograms of hierarchical clustering analysis of the 4-dimensional multidimensional scaling solutions for (A) controls, (B) patients treated for congenital bilateral cataracts, and (C) visual controls.** We identified clusters using a data driven approach. Emotion categories were represented by different colors. Intensities of the emotions (%) were labeled with numbers.

To provide a complementary view of the similarity judgments to the MDS solutions, we ran hierarchical clustering on similarity ratings. We identified clusters using a data driven approach, where an algorithm implements an adaptive, iterative process of cluster decomposition and combination and stops when the number of clusters becomes stable (Langfelder et al., 2008). As shown in Figure 5A, for the control group, different expression categories formed two clusters. One cluster included (happy, surprised, fearful and neutral expressions). The other cluster included (sad, disgusted and angry expressions). For the patients (Figure 5B), there were also two clusters. One cluster included (happy and surprised expressions). The other cluster included (fearful, sad, disgusted, angry, and neutral expressions). Patients differed from the controls in two ways. The first difference is that neutral is clustered with negative expressions for the patients, while for the controls, neutral is clustered with happy and surprised expressions. This result suggests that patients perceived neutral as more similar to negative expressions, while controls perceived neutral as more similar to positive expressions. The second difference is that for the patients, fearful expressions were clustered with the other negative expressions, while for the controls, fearful expressions were clustered with surprised and happy expressions. This result suggests that patients are less influenced by the physical similarity between fearful and surprised expressions (in the current stimulus set, fearful and surprised expressions were most similar based on pixel-wise cross-correlation) than the controls and perhaps more influenced by the pleasantness of the implied emotion.

Interestingly, these same two differences occur in the comparison between the perceptual structures of similarity judgments of facial expressions in visually normal 7-year-olds and adults (Gao et al., 2010). Unlike adults, 7-year-olds perceive neutral as more similar to negative expressions and they perceive fearful expressions as more similar to other negative expressions rather than to surprised expressions. However, such differences are absent at age 14. Therefore, the clustering results suggest that the development of the perceptual structure of facial expressions in patients treated for bilateral congenital cataracts may follow a normal trajectory but level off at an immature stage.

### Perceived distance vs. physical distance

In our previous study (Gao et al., 2010), perceived similarity of emotions did not map linearly to physical similarity. Instead, a pair of expressions of higher intensity (e.g., 70 and 90% anger) was perceived as more similar than a pair of expressions of lower intensity with the same physical difference (e.g., 30 and 50% anger), as would be predicted from the evidence for Weber fractions in many other aspects of perception (Fechner, 1860; von Helmholtz, 1867). Here, we tested if this pattern was replicated in the current sample of visually normal adults and if such a pattern was shown in the patient group. For each participant, we calculated the mean perceived distance of a 20% change in intensity in the two intensity ranges (50–70 and 70–90%) by averaging across the six expression categories, since the current small sample size was inadequate to include expression category as a factor in the analysis. A mixed model ANOVA with group (patients vs. control) as between subject variable and intensity range (50–70 vs. 70–90%) as a repeated measure revealed a significant main effect of intensity range [*F*_(1, 18)_ = 4.38, *p* = 0.05] with no other significant main effect or interaction. The same physical difference in intensity (20%) was perceived as more similar for the higher intensity pairs (70–90%) than for the lower intensity pairs (50–70%) by both patients and controls. This non-linear relationship between physical difference and the perceived difference has been demonstrated in the categorical perception of faces (e.g., facial expressions: Etcoff and Magee, 1992; Calder et al., 1996; facial identity: Blanz et al., 2000) and in other perceptual domains (e.g., color perception: Davidoff, 2001; see Goldstone and Hendrickson, 2010 for a review).

### Correlation with duration of deprivation and visual acuity

Besides comparing the patients and the controls at a group level, we also investigated how differences in the duration of visual deprivation and visual acuity are associated with the individual MDS structures of the patients. To do so, we compared the 4-dimensional MDS solution of each patient to a model MDS solution calculated based on the average similarity ratings of all the controls. We rotated and scaled the solution of each patient to best match the model solution (Procrustes analysis) and used the least squared error between the patient solution and the control solution as a measure of similarity. We measured the Pearson correlation between the duration of deprivation (the shorter duration if there was a difference between the two eyes) and the similarity measure. Within our small sample, there is no relationship (*r* = 0.01, *p* = N.S.). The similarity measure was also not correlated with log acuity of the better eye (*r* = −0.11, *p* = N.S.).

## Discussion

The results from the control group replicate the major findings from our previous study of adults with normal vision (Gao et al., 2010): two overarching clusters were formed. One cluster included (happy, surprised, fearful, and neutral expressions). The other cluster included (sad, disgusted, and angry expressions). As in previous studies, MDS reveals additional structure in the form of four underlying dimensions that appear to represent intensity, arousal, pleasure and potency (Russell, 1980; Russell and Bullock, 1985, 1986; Gao et al., 2010). Two of these underlying dimensions (pleasure and arousal) are already evident at 2 years of age (Russell and Bullock, 1986) but the structure is not fully adult-like even at age 14 (Gao et al., 2010).

Here the patient group showed systematic structure in their judgments of the similarity of the basic emotions presented at three levels of intensity, and their MDS solutions were largely interpretable. These findings suggest that they are able to perceive moderately intense expressions and decode their emotional meaning in a fairly normal way. However, there were differences between the patients and the control group: patients grouped neutral with negative expressions rather than with happy expressions; they grouped fear with the other negative emotions instead of the more physically similar expressions of surprise; the fourth dimension revealed by MDS was not interpretable; there was no evidence of a dimension of arousal (maximally distinguishing anger from sadness); and they made judgments that varied more within the group than was true for the control group, as evidenced by higher stress values and more dispersed weightings on all four dimensions. In fact, the variability in the patient group was higher than that we observed in typical 14-year-olds, the youngest children tested with the same method (Gao et al., 2010). Their clustering of neutral with negative emotions resembles the groupings made by typical 7-year-olds tested with a similar method (Gao et al., 2010) but the underlying dimensions from the MDS solution do not resemble those observed in typical children or adults at any age: even by age 2 (youngest tested) children's judgments reveal an underlying dimension of arousal. Thus, early visual input appears to be necessary for the normal representation of facial expressions to emerge later in childhood. The absence of a correlation with duration of deprivation suggests that as little as 2 months of visual deprivation from birth is sufficient to prevent later normal development.

### Control experiment

As a result of the early visual deprivation, the patients in the current study have reduced visual acuity along with, in many cases, other secondary visual problems such as nystagmus. One concern is that patients with nystagmus may not have perceived stable images of the face stimuli and such instability might have influenced their similarity judgments of emotions in the current study, although we note that the results were similar for patients with and without nystagmus perhaps because the effect of nystagmus can be reduced by the visual brain, which can correct for consistent patterns of image instability (Melcher, 2011).

Another concern is that reduced acuity prevented the patients from seeing critical fine details in the face stimuli and that the lack of correlation with acuity arose because every patient had insufficient acuity to perform the task like controls. However, that seems unlikely because cataract-reversal patients like those tested here have normal contrast sensitivity for low and mid spatial frequencies (up to 2–3 cycles/°) and can see higher spatial frequencies at increased contrast up to their acuity cut-off (Ellemberg et al., 1999), which averaged 15 cycles/deg in the current cohort. Adults with normal vision do not use spatial frequencies near their acuity cutoff in decoding facial expressions. For example, with this stimulus set, we have shown that adults with normal vision use mid-spatial frequencies (centered on 16 cycles/face width or 2.8 cycles/°) to discriminate facial expressions of happy, sad, fear, and anger (Gao and Maurer, 2011). Even for the facial expression whose recognition relies on relatively high spatial frequencies (e.g., fear, Smith and Schyns, 2009), the most critical spatial frequency is below 60 cycles per face width (10.7 cycles/° at our testing distance), a value lower than the median acuity of the better eye in the current sample (15 cycles/°).

To investigate empirically the potential influence of nystagmus and reduced visual acuity on the perceived similarity among our facial expression stimuli, we tested another control group of adults with normal vision on a set of faces with reduced image quality: from here on, we refer to this new control group as the visual control group. Specifically, we low-pass filtered the face stimuli to remove high spatial frequency content that would not be available to patients with reduced visual acuity, using the median acuity in the better eye (i.e., 20/40) as a cutoff. We also jittered the positions of the stimuli to simulate unstable retinal images caused by horizontal nystagmus, the predominant direction of nystagmus in the patient group.

### Participants

Ten Caucasian adults (mean age = 26.3 ± 6.0 years, range = 21–35 years, 5 females) with normal or corrected-to-normal vision participated in the study. All participants provided informed consent.

### Stimuli

The stimuli were the same as in the first experiment, except that spatial frequencies above 87 cycles/face width were removed using an ideal filter. We calculated the cutoff frequency based on the median value of the visual acuity (20/40) of the better eyes in the patient group. The finest spatial frequency one can see with an acuity of 20/40 is 15 cycles per degree. Since each face spans 5.8° of visual angle at a distance of 60 cm, the cutoff frequency is 87 cycles/face width.

### Procedure

**Figure 6:**
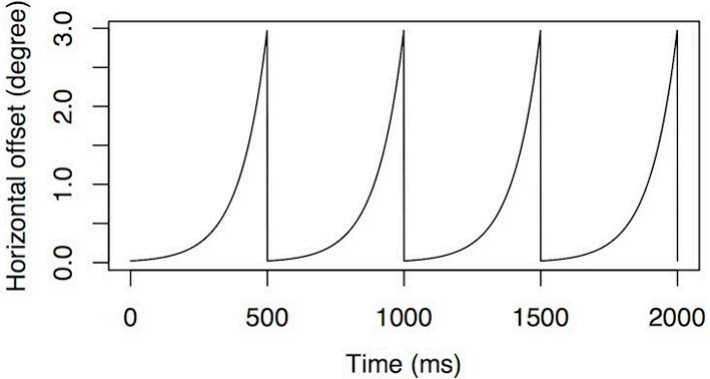
**Simulated nystagmus waveforms.** An accelerating velocity exponential slow phase with amplitude of 3° of visual angle at 2 Hz.

We used the same procedure as in the first experiment, except that the horizontal position of the face images was constantly jittered according to a periodic exponential function shown on Figure 6. The function has a maximal horizontal displacement of 3° of visual angle and a frequency of 2 Hz.

### Results and discussion

We began by running split-half correlation analysis on the similarity matrices (Figure 2C) as described earlier. The within group correlation of the visual control group (*r* = 0.87) and the correlation between the visual control group and the original control group (*r* = 0.82) were both high and not different from the within group correlation of the original control group (*r* = 0.90, 95% *CI* = 0.79–0.97, *p* = N.S.). However, the correlation between the visual control group and the patient group (*r* = 0.75, 95% *CI* = 0.61–0.83) was significantly lower than the within group correlations of either control group (*p*s < 0.05). The results suggested that the similarity rating data of the visual control group was not differentiable from the original control group, while the patient group differed from both control groups.

MDS analysis suggested a four dimensional solution would explain the data of the visual control group optimally (Figure 3). By correlating the MDS solutions with the independent ratings we identified three dimensions representing pleasure (dimension 1), arousal (dimension 2), and potency (dimension 3) (Table 2). An intensity dimension was not revealed by the correlations with the independent ratings. However, the fourth dimension in the MDS solution of the visual control group may capture the within emotion category intensity (Figure 4C). To provide an objective measure of the similarity of the MDS solutions between the visual control group and the original control group and between the visual control group and the patient group, we ran a Procrustes analysis. In this analysis, we rotated and scaled the MDS solutions of the visual control group and the patient group to fit the MDS solution of the original control group and used the residual of the fit as a measure of similarity, so that a smaller residual value would represent a better fit. To set a standard, we derived the 95% confidence interval of the fit between the MDS solution of the original control group and the MDS solutions of bootstrapped samples of the original control group (95% *CI* = 0.021–0.176), The fit between the visual control group and the original control group (0.139) was within this standard while the fit between the patient group and the original control group (0.185) was outside of this confidence interval. The results suggested that the MDS solution of the visual control group was not statistically different from the original control group (*p* = N.S.), but the MDS solution of the patient group differed from the original control group statistically (*p* < 0.05). Hierarchical clustering also revealed that the visual control group had a very similar clustering to the original control group (Figure 5C). For the visual control group, the facial expressions formed two overarching clusters, one including (anger, disgust, and sadness), and another one including (neutral, happiness, fear, and surprise).

These complimentary measures provide evidence that despite the fact that the image quality for the visual control group was degraded by low-pass filtering and position jittering, the perceived similarity structure of facial expressions in the visual control group was not affected. The highly similar structures between the two control groups reported in the current study as well as the similarity of both with the structure of adults in the previous study (Gao et al., 2010) suggest that in visually normal adults the underlying structure of facial expression is very robust. The control results suggest that the differences in the patients' structure are unlikely to have arisen from image degradation during the test resulting from reduced acuity and/or nystagmus.

## General discussion

When early visual input was missing during the first 2–10 months of life because of bilateral congenital cataracts, many aspects of the processing of facial expressions appear nevertheless to develop normally. In a previous study of this cohort (Geldart et al., 2002), patients had normal accuracy in matching intense facial expressions posed in photographs of different individuals. In the current study, when the same cohort of patients judged perceived similarity among emotions, as a group, they showed a systematic underlying structure of the representations of emotions, with underlying dimensions representing pleasure, potency, and intensity. Developmentally, these dimensions emerge before adolescence. However, a dimension representing arousal is not obvious in the structure of the patients. Clustering analysis on the MDS solutions also revealed differences between patients' structure and the structure of normal adults. Fear and neutral were clustered with anger, sadness, and disgust in the patients. In contrast, fear and neutral were clustered with happiness and surprise in normal adults. The pattern of clustering seen in the patients resembles what we have found in 7-year-olds. However, this is not a simple delay in development because an arousal dimensions is already seen in the structure of 2-year-olds (Russell and Bullock, 1986), but not in the structure of the current sample of patients. On the other hand, a potency dimension is seen in the structure of the patients, despite the fact that it arises late during typical development (Gao et al., 2010). We can offer no explanation of why the arousal dimension was the one missing and no interpretation of patients' unlabeled dimension. It would be interesting for future studies to investigate how arousal is perceived by the patients with a more direct method (e.g., rating the emotions explicitly on an arousal dimension) than the one used in the current study.

The current findings add to the literature a new piece of evidence about the role of experience in fine-tuning the representation of expressions. The effect of early visual deprivation is likely to operate through an experience expectant mechanism. Typically developing infants are expected to see patterned visual input including faces in a typical environment. The normal visual input may setup (or preserve) neural architecture for later refinement dedicated to the processing of facial expressions. Since the refinement of the representation of facial expressions continues into late childhood, the effect of early visual deprivation on the later development is an example of a sleeper effect (Maurer et al., 2007).

The patients' deficits are unlikely to have been caused solely by associated conditions such as poor acuity and contrast sensitivity, nystagmus, eye misalignment, or glaucoma. Patients were tested binocularly and all had acuity of at least 20/80 in one eye (7.5 cycles/°, 43.5 cycles/face width). Moreover, early binocular deprivation largely spares contrast sensitivity for mid-spatial frequencies (Birch et al., 1998; Ellemberg et al., 1999), the range used by typical adults when making judgments of facial expressions, even those of low intensity (10 cycles/face width, with a bandwidth of two octaves, Gao and Maurer, 2011). Despite their acuity deficits, patients treated for bilateral congenital cataract are also normal in discriminating small differences in the shape of the mouth and eyes in upright faces (Mondloch et al., 2010) and hence likely to be able to pick up information from those regions that signals emotion. In addition, in the control experiment, visually normal adults' perceived structure of emotions was not affected even when the faces were blurred and their positions jittered. However, we cannot totally rule out the possibility that degraded visual input post-treatment may have a cumulative effect on the patients' perceived structure of emotions. Nonetheless, the pattern of results from patients was not related systematically to any of these variables (see Table 1), at least within this small sample. The deficits are also unlikely to represent merely a delay in development because they were present even in patients more than 30 years old.

There are a number of limitations in the current study. The size of the patient sample that we were able to recruit was small, because of the rarity of the condition. With a larger sample, future studies may be able to identify the dimensions that were not interpretable in the current patient sample. We used only one female model in order to reduce one possible source of variation and because in our previous study with one female and one male model we found no differences in the results for the two models (Gao et al., 2010). Nonetheless, future study should investigate whether the effects found here generalize to other models and whether they differ specifically for male vs. female models, possibly as a result of the different facial morphology of male and female faces. Another limitation is that, although the MDS approach is useful in mapping the underlying structure of the perceived similarities among emotions, its interpretation is subjective and makes it difficult to make quantitative comparisons between patients and controls. In future studies, it would be useful to implement more quantitative measures, such as the Bubbles technique (Gosselin and Schyns, 2001), which could be used to examine whether the same features are used by the patients and the controls in discriminating among facial expressions. It would also be useful to collect categorical responses or ratings of emotion intensity for discrete emotion categories (e.g., Adolphs et al., 1994) as another method for mapping the underlying structure of emotions. This method would also allow a more quantitative way of comparing the patients with the controls (e.g., train a Bayesian classifier with the data from the controls and test it with the data from the patients). Future study can also investigate how the patients categorize facial expressions using a forced choice paradigm. Confusion matrices arising from a forced choice procedure would also provide information about the relationship among emotion categories.

In conclusion, early visual experience appears to be necessary, not only for the normal development of sensitivity to facial identity (Le Grand et al., 2001, 2003, 2004; Geldart et al., 2002; Mondloch et al., 2003b; Robbins et al., 2010, 2012; de Heering and Maurer, 2012), but also for the development of the complex structure underlying normal adult representation of emotions. Future studies might investigate whether sensitivity to other facial attributes, such as attractiveness and deviations of eye gaze, are also affected, as well as whether the integration of facial attributes (e.g., eye gaze and expression) is compromised.

## Acknowledgements

This research was supported by grants from the Canadian Natural Sciences and Engineering Research Council (9797) and the James S. McDonnell Foundation (collaborative activity award). We thank Sally Stafford for help in testing the patients.

### Conflict of interest statement

The authors declare that the research was conducted in the absence of any commercial or financial relationships that could be construed as a potential conflict of interest.
